# Data- and knowledge-based modeling of gene regulatory networks: an update

**DOI:** 10.17179/excli2015-168

**Published:** 2015-03-02

**Authors:** Jörg Linde, Sylvie Schulze, Sebastian G. Henkel, Reinhard Guthke

**Affiliations:** 1 Research Group Systems Biology / Bioinformatics, Leibniz Institute for Natural Product Research and Infection Biology - Hans-Knöll-Institute, Beutenbergstr. 11a, 07745 Jena, Germany; 2 BioControl Jena GmbH, Wildenbruchstr. 15, 07745 Jena, Germany

**Keywords:** gene regulatory networks, modeling, reverse engineering, network inference, prior knowledge, RNA-Seq

## Abstract

Gene regulatory network inference is a systems biology approach which predicts interactions between genes with the help of high-throughput data. In this review, we present current and updated network inference methods focusing on novel techniques for data acquisition, network inference assessment, network inference for interacting species and the integration of prior knowledge. After the advance of Next-Generation-Sequencing of cDNAs derived from RNA samples (RNA-Seq) we discuss in detail its application to network inference. Furthermore, we present progress for large-scale or even full-genomic network inference as well as for small-scale condensed network inference and review advances in the evaluation of network inference methods by crowdsourcing. Finally, we reflect the current availability of data and prior knowledge sources and give an outlook for the inference of gene regulatory networks that reflect interacting species, in particular pathogen-host interactions.

## Introduction

One characteristic of life is that living organisms constantly adapt to environmental changes (Koshland, 2002[79]). Higher organisms may use their brain for long term reactions or reflexes as nearly instant reactions in response to stimuli. On a molecular basis, microorganisms as well as cells and tissues of higher organisms sense environmental changes (Groisman and Mouslim, 2006[52]). This information is then transmitted into the cells and processed which finally leads to a reaction of the cells. Sensing, transmitting and processing of the information is performed by complex molecular interactions (Miller and Bassler, 2001[112]). While our knowledge of these interactions is still limited, it is obvious that errors in information processing may lead to diseases (Follo et al., 2015[42]; Compston and Coles, 2008[27]; Wang et al., 2012[166]; Glocker et al., 2006[47]).

Systems biology is a research area which aims to understand living systems as a whole, instead of focusing on single biological entities (Ideker et al., 2001[71]). Systems biology is often (but not exclusively) connected with omics. Here, researchers characterize and/or quantify (nearly) all biological molecules of a specific type which allows us to study the complete picture of the system. For example, transcriptomics measures the abundance of all transcripts in a sample, while proteomics measures the abundance of all proteins. One way of describing biological systems are networks, i.e. graphical representations, where the nodes represent objects of interest and edges represent relations between these objects (Le Novère et al., 2009[84]). Network models do not only help to explain, understand and describe the functioning of a cell (Barabási and Oltvai, 2004[12]), but also to understand disease progression and to discover drugs (Butcher et al., 2004[20]). In gene regulatory networks (GRNs) nodes are genes and edges represent interactions between genes, such as activation or repression. Genes do not necessarily interact directly with each other. In fact, the most direct interaction is a gene coding for a transcription factor (TF) which binds to the promoter region of another gene and regulates its expression. A gene can also influence the expression of another gene more indirectly, via signaling cascades or whole pathways. For sake of simplicity these influences are also described with the word ‘interaction’.

As the underlying structure of many networks is not (completely) known, one focus of systems biology is uncovering the complex and dynamic interactions between genes (Hecker et al., 2009[66]). The research area called 'network inference (NI)' aims at the deduction of network structures utilizing high-throughput data with help of reverse engineering techniques. In most cases transcriptome data is used. NI consists of three parts: the identification of potential regulators, the prediction of target genes and the inference of the mode of interaction (e.g. activation or repression). The number of genes may vary from only two genes to full-genomic networks. A general problem in NI is the high dimensionality (thousands of genes) versus the limited number (tens to hundreds) of samples. Thus, GRN inference is underdetermined (‘curse of dimensionality’) implying that there could be many equivalent (indistinguishable) solutions (networks). Motivated by this fundamental problem, there exists a number of NI approaches, which are compared in outstanding review articles (de Jong, 2002[29]; van Someren et al., 2002[162]; Gardner and Faith, 2005[46]; Bansal et al., 2007[8], Ay and Arnosti, 2011[5]; Wu and Chan, 2012[173]; Emmert-Streib et al., 2014[35]). In 2009, our group thoroughly reviewed NI approaches with a focus on data integration (Hecker et al., 2009[66]). In the review on hand, we present an update of the former review with a special focus on novel techniques for data acquisition, NI assessment and NI for interacting species.

In what follows, we give an overview about the main NI approaches focusing on novel and updated methods introduced since 2009. During that time, the NI community has emphasized the inference of large-scale or even full-genomic networks, the integration of additional data and the combination of NI methods. The integration of data from various (omics) experiments and knowledge databases into one general model is an important challenge in systems biology (Gomez-Cabrero et al., 2014[49]). In the field of NI, additional data is often integrated with help of prior-knowledge, i.e. predicted or known interactions based on additional data or knowledge sources. Here, we give an overview of commonly used prior-knowledge sources. 

**Figure 1:**
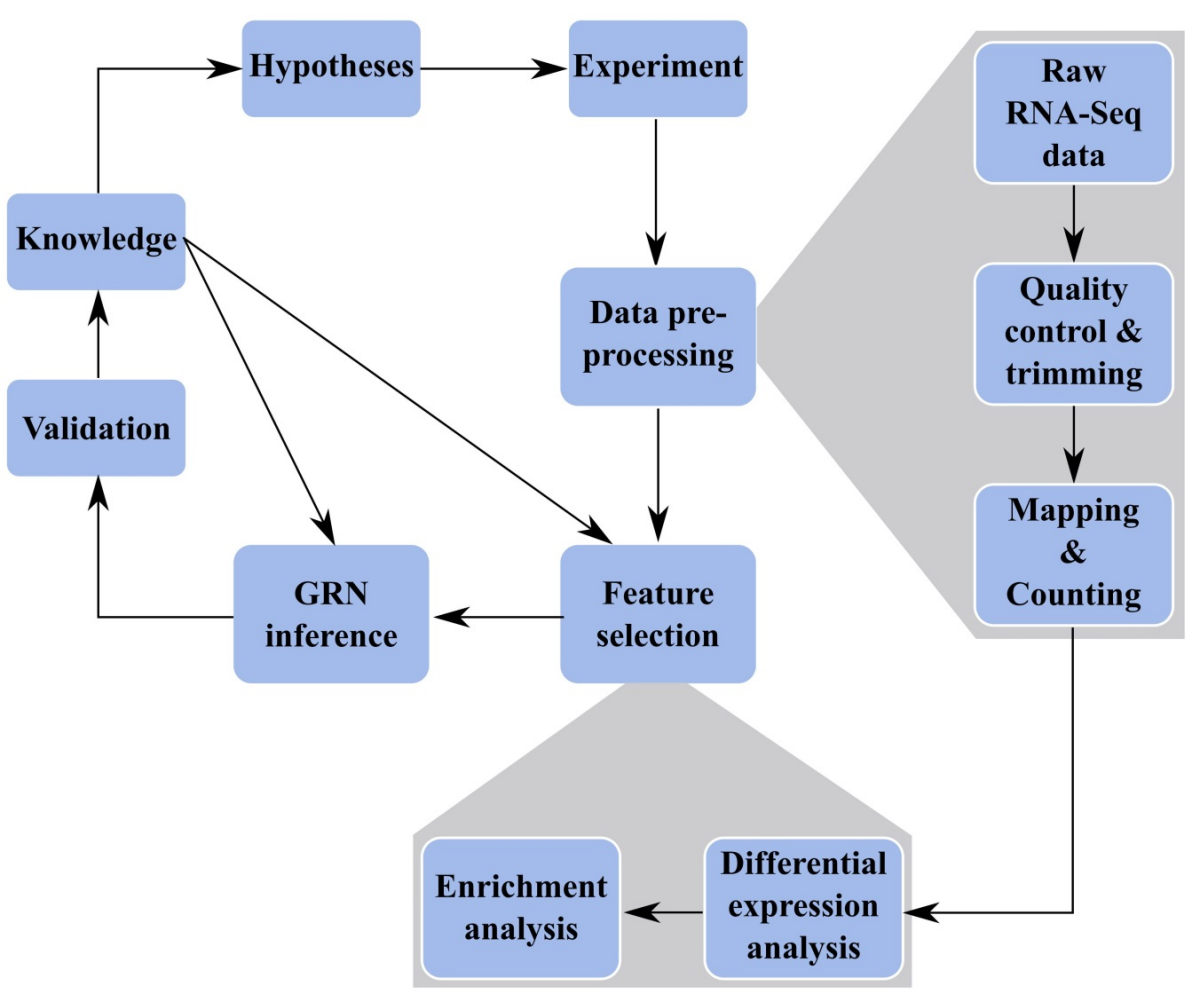
Workflow of GRN inference. Systems Biology Cycle of wet lab (experiment) and dry lab work: Experiments lead to RNA-Seq data, which need to be preprocessed and features have to be selected (more detailed steps are shown in grey boxes). A GRN is inferred for selected features. Predicted interactions are validated leading to more knowledge and new hypotheses. Both analysis of experimental data (data preprocessing and feature selection) and modeling (network inference) is supported by prior knowledge.

The advance of Next-Generation-Sequencing of cDNAs derived from RNA samples (RNA-Seq) allows to study transcriptomes with a so far unreachable depth and quality (Morin et al., 2008[116]). On the other hand, data pre-processing poses new challenges. Here, we describe a work-flow combining RNA-Seq data analysis with NI (Figure 1(Fig. 1)). In particular, the advance of RNA-Seq allows researchers to perform transcriptome studies of interacting (micro-) organisms using the same technology without having to separate RNA samples (‘dual RNA-Seq’; Westermann et al., 2012[171]). This allows to predict GRNs of organisms which interact with each other. Special interest is in pathogen-host interaction networks which we present in this review.

A particular challenge is the evaluation of predicted networks. Advances in the evaluation of NI methods by crowdsourcing within the DREAM initiative are described in this review.

## Gene regulatory network inference

**Figure 2:**
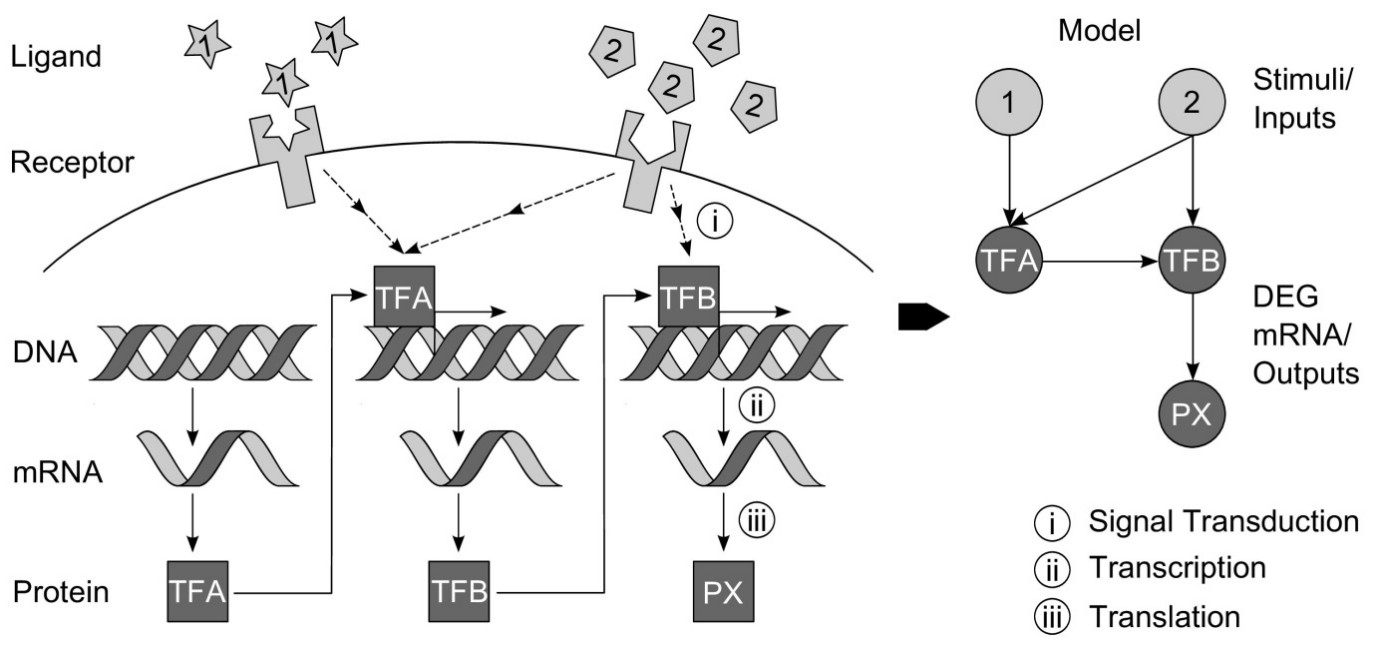
Regulated gene expression and model network representation. External stimuli (ligands binding to receptors on the cell’s surface) may trigger an alteration in gene expression. Via signal transduction, the most important regulators, the transcription factors, are influenced. They regulate the transcription of DNA to mRNA, which subsequently is translated to proteins. Those regulated biological processes can be transformed to a network model (inference), whose main nodes represent genes or their products (typically on the level of regulated transcription).

NI aims to determine the structure and parameters of GRNs. Due to various sources of perturbation, biological systems adapt gene expression and their functionality. The main biological processes and components as well as a model network representation are shown in Figure 2(Fig. 2). To fulfill complex tasks and functionalities of living systems and to adapt to various perturbations gene expression changes with respect to their amount (concentration, activity) and influences the expression of other genes. The terminus ‘Gene Regulatory Network’ (GRN) refers to components of transcriptional regulation, i.e. target genes and TF genes, specific products of gene expression allowing for a complex regulatory response. Understanding of GRNs means understanding of underlying mechanisms and the potential for targeted manipulation of biological systems (Figure 2(Fig. 2)).

GRNs can be described by mathematical network models built up of nodes describing the components of gene expression, i.e. genes or their products, and edges, i.e. connections between nodes denoting an interacting effect. Nodes and edges together form a network that is primarily defined by its structure, which means existing or non-existing connections between nodes. One important property of GRNs is the sparseness, which refers to the fact that in biological networks much less edges occur than theoretically possible, i.e. in a fully connected network (Leclerc, 2008[85]). One additional specific property is the scale-freeness of GRNs. That means the number of edges per gene follows a power law distribution: Many nodes with a low number of connections and few nodes with a high number of connections (‘hubs’) exist.

The interactions presented as edges in the graphical presentation of GRNs (Figure 2(Fig. 2)) may exhibit different strengths of interactions or different molecular mechanisms. For describing those properties mathematical models are needed. Nonzero parameters of these models are represented in the graph. By identifying the nonzero parameters of a model, NI reveals the structure of the system. Additionally, parameter optimization methods are applied to estimate the exact value of the parameters which can be interpreted as the strength of the interaction. To infer underlying GRNs of a biological system, NI methods use data measuring the gene expression intensity and/or the abundance/activity of proteins. In most cases transcriptome data is used. Advanced methods need to know the variance of a measurement (via replicates) and temporal resolution. Different assumptions on underlying processes, prior knowledge about molecular mechanisms, available mathematical methods and many more constraints led to the development of different NI methods. Most of the presented NI methods consider transcriptional regulation, measure mRNA levels which shall reflect the major behavior of regulatory processes and not the mechanisms but only the existence of interactions is the focus of NI, i.e. relatively large data sets are used for an automatic top-down modeling.

## RNA-Seq data analysis for gene regulatory network inference

### Data requirements

NI often relies on transcriptome data. Common technologies for high throughput transcriptome studies are microarrays and RNA-Seq where microarrays have been frequently used. Advantages and disadvantages regarding their applications, costs and time consumption, sensitivity and dynamic range of detection, challenges in data analysis and data storage have been outlined in various publications (Mantione et al., 2014[98]; Malone and Oliver, 2011[97]; Wang et al., 2009[168]). In general, RNA-Seq allows researchers to study transcriptomes with a so far unreachable depth and quality (Morin et al., 2008[116]). Furthermore, RNA-Seq allows to study the transcriptome of non-model organisms, since the expensive design and spotting of arrays is not necessary. In fact, RNA-Seq may be performed even without having the genome sequence at hand (CUFFLINKS (Trapnell et al., 2012[158]), TRINITY (Haas et al., 2013[59]). Obviously, in future there will be more NI approaches based on RNA-Seq data. For this reason, we summaries major steps in RNA-Seq based network inference here.

When studying interacting species it is of interest to monitor the transcriptome of all present species. Microarrays can be utilized, when it is known which species interact and microarrays are available for these species. These limitations do not apply for ‘dual RNA-Seq’, where RNA samples of two (all) species are sequenced together and transcripts are separated in silico. This also prevents a possible transcriptional change caused by the experimental separation of species. In the following we summarize RNA-Seq data properties, RNA-Seq platforms that open new opportunities in GRN inference as well as ‘dual RNA-Seq’.

RNA-Seq is a powerful technology for transcriptome profiling, but the understanding of data properties is incomplete and standard protocols for data generation and analysis are lacking. Eukaryotic total RNA consists of ~80 % rRNA, ~15 % tRNA and only a small portion of mRNA (Lodish et al., 2000[93]). To increase the informative output of an RNA-Seq study, samples can be depleted for rRNA or enriched for RNA species of interest (e.g. polyadenylated RNAs) (Sims et al., 2014[147]). Nevertheless, this results in a loss of information and has to be considered with caution especially when dual RNA-Seq is carried out. To mention a single example, let's assume one wants to study the transcriptome of human cells infected with bacteria. In bacteria most mRNAs are not polyadenylated, only when they are tagged for degradation whereas in human cells mRNAs are polyadenylated. Consequently, an enrichment for polyadenylated RNAs would result in a misleading RNA sample (Westermann et al., 2012[171]). Furthermore, Lahens et al. (2014[83]) concluded that rRNA depletion introduces a bias in coverage. 

Given a fixed budget, a trade-off between the number of sequenced reads and the number of replicates is needed. Based on mRNA enriched samples of the human cell line MCF7, Liu et al. (2013[91]) found that lower sequencing depth, but a higher number of biological replicates increases the power and accuracy to detect differentially expressed genes. Sequencing a number of reads resulting in more than 10 million mapped reads led to a significantly smaller improvement than generating more replicates instead. Nevertheless, the sequencing depth has to be determined taking the research question into account, because an accurate identification of lowly expressed transcripts requires a sufficient amount of reads (Sims et al., 2014[147]). 

Another challenge arises from the nature of the human genome – approximately 50 % is constituted of repetitive elements. Typically, only 70-80 % of short reads map uniquely to the human genome depending on read length and availability of paired-end reads. Methods for assigning ambiguous reads have been briefly reviewed by Treangen and Salzberg (2011[159]). 

Recently, the question whether RNA-Seq is reproducible was addressed. The GEUVADIS consortium (Genetic European Variation in Disease, a European Medical Sequencing Consortium) found small technical variation in samples sequenced with Illumina HiSeq2000 using the exact same protocols (‘t Hoen et al., 2013[154]). Within the SEQC/MACQ-III (Sequencing Quality Control) project different sequencing platforms across multiple laboratory sites and analysis pipelines were examined for the detection of differential expression. They found reproducibility when filters for p-value, fold-change and expression level were applied (SEQC/MAQC-III Consortium 2014).

Multiple sequencing platforms have been developed over 10 years of next-generation sequencing research. All second generation platforms, i.e. 454, SOLiD and Illumina, are light-based capturing a fluorescence signal. New approaches, termed ‘third generation’ are emerging such as the light-based PacBio, pH-based Ion Torrent and current-based Oxford Nanopore. A good overview over second and third generation sequencing platforms is outlined in Liu et al. (2013[91]). One advantage of third generation platforms are steadily increasing read lengths. Applying the PacBio system, read lengths > 1400 bp (Mosher et al., 2014[117]), were reported recently. A first paper about the application of the Oxford Nanopore sequencer reports average read lengths of 5 kb, but concludes that a dramatic decrease of error rates is required (Mikheyev and Tin, 2014[111]). Increasing read lengths of third generation technologies will help to overcome problems such as ambiguous mapping, but second generation platforms are still dominating in application.

### RNA-Seq data trimming

Typically, the first step of RNA-Seq data analysis (Figure 1(Fig. 1)) is clipping of sequencing adapters and removing low quality bases (‘trimming’), followed by read mapping and counting. Recently, nine trimming algorithms were evaluated on Illumina RNA-Seq data sets (Del Fabbro et al., 2013[30]). They found comparable performances of all tools (ConDeTri, Cutadapt, ERNE-FILTER, FASTX, PRINSEQ, Sickle, SolexaQA, SolexaQA-BWA, Trimmomatic) applying them to a high quality data set of Arabidopsis thaliana. Given a lower quality data set of Homo sapiens, SolexaQA performed best in terms of keeping the most reads and aligning a high percentage of them. In comparison, other tools such as FASTX did not show good performance. Some trimming tools have been developed for specific platforms, such as Trimmomatic (Bolger et al., 2014[18]) for Illumina data. Besides, trimming tools include different properties regarding e.g. adapter removal or application to paired-end reads (Jiang et al., 2014[75]).

#### Read mapping

A very important preprocessing step which has great influence at down-stream analysis is the alignment of reads to the reference genome (mapping). In a recent review, ten alignment tools were evaluated regarding multiple properties such as alignment yield, spliced alignments, mismatches and accuracy (Engström et al., 2013[36]). The aligners MapSplice, GSNAP, GSTRUCT and STAR were evaluated as favorable tools, even though the latter three reported many false exon junctions. It was concluded that TopHat2 is an effective tool, although only 84 % of reads were aligned in comparison to 90 % of reads aligned with MapSplice. Alignment tools like TopHat-Fusion, FusionSeq or SplitSeek have been developed to align reads generated from cancer cells, for which fusion genes caused by rearrangement events (e.g. chromosome breakage and rejoining) are common (Treangen and Salzberg, 2011[159]). When analyzing dual RNA-Seq data, the genomic landscape of interacting species has to be taken into account to determine alignment tool parameters. For example, potential host organisms such as human and mouse have a high percentage of intron-containing genes. On the other hand, the percentage of intron-containing genes in pathogenic fungi varies a lot, e.g. Candida glabrata has ~1.5 % and Cryptococcus neoformans has ~97 % intronic genes (Ivashchenko et al., 2009[74]), whereas bacteria do not have introns at all. 

### Counting

After the mapping step, the number of reads assigned to a feature (e.g. exon, transcript, gene) has to be counted to estimate the expression level. This data needs to be corrected for biases, but standard approaches usually depend on the feature length and the non-uniform distribution of reads to features. The new counting approach maxcounts is based on the maximum of the per-base counts and claims to reduce biases in RNA-Seq data (Finotello et al., 2014[40]). So far, maxcounts is only applicable to exon-level. Usually, counting on transcript- or gene-level is of interest for GRN inference for which the very fast featureCounts tool can be applied (Liao et al., 2013[87]).

### Differential expression analysis

The main issue of small- and medium-scale GRN inference is the feature selection, i.e. the identification of the ‘most important’ genes or proteins of interest for a certain system or process. Identification of differentially expressed genes is an important step for feature selection, i.e. to narrow down the number of network nodes. 

For RNA-Seq data, various statistical methods have been proposed and recently the performance of the common tools Cuffdiff2, DESeq and edgeR were compared by Zhang et al. (2014[181]). It was recommended to apply DESeq and edgeR preferably to Cuffdiff2, especially when sequencing depth is low (< 10M). The more conservative DESeq detects less differentially expressed genes and shows a lower false positive rate than edgeR. On the other hand, edgeR is more liberal and tolerates unbalanced library sizes and low sequencing depth. Soneson and Delorenzi (2013[148]) evaluated 11 differential expression analysis tools and found similar results regarding DESeq and edgeR. Furthermore, none of the 11 tools performed best under all circumstances and they provide a short overview about the main finding for every tool. Last year, Love et al. (2014[95]) released the updated DESeq2 and compared DESeq2 to six other differential expression analysis tool including DESeq. They found that all algorithms control the false positive rate, whereas DESeq2 is less conservative than DESeq and Cuffdiff2 and more conservative than edgeR, voom and SAMseq.

### Gene enrichment analysis 

Another option for feature selection is Gene enrichment analysis. Here, genes are grouped based on their function or biological process. The user may identify processes mostly enriched with DEGs and focuses NI only on those genes. In 2009, Huang et al. (2009[69]) listed 68 enrichment tools, classified them in three group and highlighted properties and limitations of each group. Afterwards, more enrichment tools have been published, e.g. KOBAS (Xie et al., 2011[174]). KOBAS 2.0 incorporates knowledge across 1,327 species from 5 pathway databases (KEGG PATHWAY, PID, BioCyc, Reactome and Panther) and 5 human disease databases (OMIM, KEGG DISEASE, FunDO, GAD and NHGRI GWAS Catalog). For other species not or only weakly represented in these databases, there are more specific tools. For instance, the online resource and web tool FungiFun 2.0 was developed for functional analysis of lists of fungal genes and proteins (Priebe et al., 2015[132]). Most enrichment tools assume, that all genes are equally likely to be selected as differentially expressed. Contradictory to this assumption Oshlack and Wakefield (2009[126]) found that long (or highly expressed) transcripts are more likely to be detected as differentially expressed, which also affects enrichment analysis. GOseq is one of the tools that integrates a correction for this selection bias (Young et al., 2010[179]; Rahmatallah et al., 2014[137]).

## Basic inference methods

After summarizing how to gain raw data for NI, this chapter gives an overview of basic NI approaches. Please note, that most approaches do not only work on transcriptome data but might also be applied on other (omics) data. 

Inference algorithms can be classified by their major properties (Table 1(Tab. 1)). These are (i) the underlying method, (ii) the result, (iii) the directionality of interactions, (iv) the consideration of dynamics and (v) the integration of prior knowledge (PK), i.e. putative or known interactions based on additional data sources such as literature. 

(i) The underlying method or framework describes the key aspect that characterizes the inference approach and can be: (a) Boolean modeling, (b) probabilistic modeling, (c) Information theory-based methods (Mutual Information), (d) (linear) regression and (e) (complex) optimization. These main methods will be described in the subsequent paragraphs. 

(ii) The result strongly depends on the selected method and can for example be a Bayesian network, a correlation network, a graphical model or a mathematical model consisting of algebraic or differential equations.

(iii) The model network graph may contain directed or undirected edges, i.e. in the former case cause and effect are clearly distinguished while in the latter case there is rather a general relationship. 

(iv) Whether the resulting model is dynamic, i.e. the state of the network at a certain time point also depends on its state at former time points) or static mainly depends on the consideration of time series or steady state data. 

(v) The integration of PK has been shown to improve the reliability of predicted novel interactions (Hecker et al., 2009[66]; Greenfield et al., 2013[50]; Isci et al., 2014[73]; Hasegawa et al., 2014[63]; Olsen et al., 2014[125]). Since reliable information about the experimentally verified interaction is increasing, a NI methods integrate PK in different ways most nowadays (see below section ‘Integration of prior knowledge’). One major difference is whether PK is softly integrated, i.e. the NI method may neglect an interaction within the list of PK if it contradicts the measured data and model assumptions.

Further properties of NI methods are the (vi) non-linearity or linearity, (vii) the explicit consideration of stimulation, (vii) the consideration of stochastics and application of probabilities, (viii) the network size, (ix) the number of required data and (x) the availability as a software tool. 

(vi) Mathematical models may be linear or non-linear depending on (a) how detailed and realistic molecular mechanisms are described in the model and (b) whether only the behavior in the neighborhood of a perturbed (steady-state) operating point is considered. 

(vii) Although the change in gene expression is caused by one or more (external) stimuli only few methods do consider them explicitly. 

(viii) The application of probabilities is based on the information that repeated measurements have the property to follow certain distributions originating from stochastic processes of the biological and/or technical system (measurement method). Finally, the network model size strongly correlates with the available data and assumptions that condense that data, e.g. by clustering or focusing on certain pathways.

The basic methods will be described in the following.

### Boolean Modeling

At each node (gene) of the dynamic networks resulting from Boolean modeling, the discretized input values are transformed to an output value by Boolean rules (operators) of which AND, OR and NOT are the simplest and most widely used ones. Boolean networks were first introduced by Kauffman (1969[77]), but have later been used as models of GRNs (Akutsu et al., 1999[2]; Martin et al., 2007[105]; Eduati et al., 2010[33]), in particular by the REVEAL algorithm (Liang et al., 1998[86]), a method combining Boolean modeling and information theory elements (see below). The advantage of Boolean networks is their simplicity while drawbacks are the necessity of discretizing the continuous expression values and the limited coverage of real mechanisms given by the Boolean operators.

### Probabilistic Modeling / Bayesian Networks

Probabilistic modeling is not restricted to infer GRNs, but is a common class of algorithms in that field. Most popular, probabilistic modeling infers Bayesian Networks (BN) in which the expression of each gene is considered to be a random variable following probability distributions. This major aspect also shows one of the disadvantages: the need for many data to determine the conditional probabilities. Major advantages are their ability to find hidden variables and the common and easy integration of prior knowledge. BNs are typically displayed as directed graphs that can be either static or dynamic. The latter does not follow the concept of feedback but describes the propagation of information from one time step to the next. There exist major toolboxes and packages like BNT (Bayes Net Toolbox) (Murphy and Mian, 1999[120]), BANJO (Hartemink et al., 2001[62]), ebdbNet (Rau et al., 2010[138]) and the family of BMA methods (Bayesian Model Averaging) (Yeung et al., 2011[176]; Lo et al., 2012[92]; Young et al., 2014[180]). Methods of probabilistic modeling are also described by Friedman et al. (2000[43]), Perrin et al. (2003[128]), Markowetz et al. (2005[104]) and Ernst et al. (2007[37]).

### Mutual Information / Information Theory Models

Information theory was applied to develop inference methods for GRN by using mutual information. This term measures the statistical dependency between the (discrete) states of two random variables, which represent the expression intensities of two genes. Inference methods then generate correlation-like undirected graphs containing this dependency information. Several algorithms have been developed: RELNET (Butte and Kohane, 2000[21]), ARACNE (Basso et al., 2005[13]), MRNET (Meyer et al., 2007[110]), CLR (Faith et al., 2007[38]) and C3NET (Altay and Emmert-Streib, 2010[3]). Typically, those methods are restricted to static networks, but TimeDelay- ARACNE (Zoppoli et al., 2010[182]) or tlCLR (Madar et al., 2010[96]) are able to generate directed graphs also considering time-dependent information.

### Linear Models and Regression

Mathematical models can be dynamic or steady state models depending on whether the problem formulation and solution is set up of algebraic or differential (difference) equations. A linear dynamic model can be written as
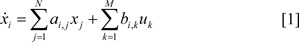


that is the change (temporal deviation) of the ith gene expression intensity χ*_i_*. The derivative χ*_i_* depends on the weighted sum of the expression intensity of all N genes (weights: a*_i,j_*, total number of genes: N) as well as potentially the weighted sum of external stimuli or inputs *u_k_* (weights: *b_i,k_*). The ordinary differential equations (ODE) [1] of all genes then form the network model. Possible variants of this description are the usage of difference equations (discrete time model) or non-linear ODEs. If the experiments, the data and thus the model are assumed to be described by steady states, the left hand side of eq. (1) is set to zero:
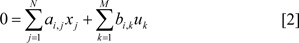


resulting in a static linear model. Sometimes this equation is re-formulated as
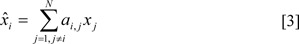


that describes the steady state expression value of the *i*th gene as the weighted sum (correlation) of all other genes neglecting the explicit formulation of stimulation. 

Linear Regression is applied to linear models described by eqs. [1-3]. By means of ordinary least squares (OLS) the solution


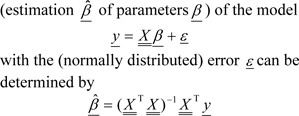
.

However, to satisfy the sparseness criterion and other properties of GRNs, methods were developed that mostly comprise the introduction of constraints. Widely used are the ridge regression (Tikhonov regularization), the LASSO (Least Absolute Shrinkage and Selection Operator) regression (Tibshirani, 1996[156]) and LARS (Least-Angle Regression) (Efron et al., 2004[34]). Multiple inference algorithms adapted to the properties of GRNs have been developed, which mostly differ in their ability to infer dynamic models: NIR (Gardner et al., 2003[45]), MNI (di Bernardo et al., 2005[32]), LARS-EN (Zou and Hastie, 2005[183]), TILAR (Hecker et al., 2009[65]), TIGRESS (Haury et al., 2012[64]) and Kulkarni et al. (2012[82]) (static models) as well as by Gustafsson et al. (2005[56]), TSNI (Bansal et al., 2006[8]), GENLAB (van Someren et al., 2006[161]), Inferelator (Bonneau et al., 2006[19]) and exTILAR (Vlaic et al., 2012[164]) (dynamic models).

### Complex Optimization

The methods of complex, mostly non-linear optimization extend the linear view of the regression mentioned before. The models are described by non-linear effects, which are the most realistic mechanisms, but many data are needed and high computational effort can be expected. Therefore, some methods have a structure and a parameter optimization step. That class of methods can be further divided into the ones that heuristically try to minimize complexity / computational effort, e.g. NetGenerator (Guthke et al., 2005[58]; Weber et al., 2013[169]) and the ones that pose no assumptions on the model, use sophisticated optimization routines but need many measurement data, e.g. DPLSQ (Nakajima et al., 2012[122]). Further optimization-based methods were described in the works by Mjolsness et al. (2000[114]), Spieth et al. (2006[150]), Nelander et al. (2008[123]) and Hasegawa et al. (2014[63]).

### Further Methods

There are further methods that often combine the above mentioned formalisms – motivated by the recent finding that no individual method performs best for all NI tasks (see section ‘Assessment of reverse engineering methods by crowdsourcing’). A method that combines Boolean and probabilistic modeling is PBN (Shmulevich et al., 2002[146]). The algorithm GeneNet (Schäfer et al., 2006[144]) is related to probabilistic modeling but uses partial correlation to infer a Gaussian Graphical Model, a network graph containing both directed and undirected edges. Küffner et al. (2012[80]) developed a method that uses analysis of variance (ANOVA) to generate non-linear correlation networks. A different approach to linear models as the ones mentioned above is the transformation into a model containing the most important components by Singular Value Decomposition (SVD) (Holter et al., 2001[67]; Yeung et al., 2002[177]) and GRN (Wang et al., 2006[167]). The GENIE3 algorithm (Huynh-Thu et al., 2010[70]) infers the GRN by applying tree-based ensemble methods for the selection of predicted interactions for each gene. The algorithm TRaCE performs an ensemble inference of GRNs, which takes into account inherent uncertainty associated with discriminating direct and indirect gene regulations from steady-state data of KO experiments (Ud-Dean and Gunawan, 2014[160]).

Probabilistic modeling and regression was combined in quite different ways by Äijö and Lähdesmäki (2009[1]) as well as Menéndez et al. (2010[108]). While the former developed an algorithm that models the regulatory functions by Gaussian processes, the latter method uses the so-called Graphical LASSO to infer undirected relationships. Yip et al. (2010[178]) presented an approach that uses two different kinds of data (knockout and perturbation), applies probabilistic modeling and optimization on differential equations, respectively and finally combines the results to directed graphs.

The Inferelator introduced by Bonneau et al. (2006[19]), lately was combined with the information theory approach tlCLR to yield dynamic models (Greenfield et al., 2010[51]). It was further combined with other methods to an iterative approach that allows the consideration of prior knowledge and finally generates a consensus networks out of a network ensemble (Greenfield et al., 2013[50]). Mutual information was also combined with an optimization-based approach resulting in the framework NIMOO (Gupta et al., 2011[54]). 

While many of the previous methods integrate several results to a final network, some algorithms that could be termed Graph Analysis focus on the elimination of false interactions (pruning of the network). Pinna et al. (2010[130]) used knockout data to establish direct cause-effect relationships and to remove unnecessary feed-forward edges. Flassig et al. (2013[41]) proposed a framework that determines an initial graph from genotype and phenotype correlations and afterwards identifies and removes indirect effects.

## Genome-wide versus small- and medium-scale networks

### Genome-wide networks

Using a holistic approach – in contrast to the reductionist approach – systems biology claims a genome-wide perspective in life sciences exploiting so-called omics data that became measurable by high-throughput techniques within the last decades (Sauer et al., 2007[143]). However, genome-wide GRN inference is rarely performed because in general the number of genes and proteins and possible interactions between them in a living organism is much greater than the number of samples and measured data. There are three approaches to tackle this ‘curse of dimensionality’:

First, the number of measured data can be moderately increased. However, increase of data by interpolation (D’haeseleer et al., 2000[31]) does not introduce additional information from the real biological system which could result in overfitted models. Thus, integration of different data, e.g. from the genome (genotyping, SNPs, TF binding sites, epigenetics), transcriptome, proteome, and interactome (protein-protein, protein-DNA interaction), in particular data from high informative experiments such as external stimulation, knock-out and knock-down experiments, is the best way to tackle the dimensionality problem. On the other hand, generation of more data is of course more expensive in different extent (Meyer et al., 2014[109]). In addition, integration of heterogeneous data is challenging and requires suitable bioinformatics tools. 

Second, knowledge and hypotheses can be introduced to restrict the degree of freedom for GRN modeling. This may be generic or specific. A widely used general assumption is the sparseness of GRNs. This reflects that not all genes and proteins are interconnected. The number of interactions is assumed to be small or only the most important (e.g. strongest) edges are inferred. Next, the number of interactions is reduced in such a manner that the complexity of the inferred network fits to the provided measurements. However, it is known, that there are regulators (e.g. transcription factors, TFs) – often called hubs – that are interlinked with many target genes, i.e. with a high outdegree. Therefore, an enhancement to sparseness is the assumption of scale-freeness (Barabási and Albert, 1999[11]). The outdegree distribution of a scale-free GRN follows a decreasing power law, i.e. the fraction *P(k)* of nodes having *k* connections to other nodes is proportional to a power term with the basis *k* and a negative exponent -γ (for a large number of nodes):

*P*(*k*) ~ *k*^-^^γ^, γ > 0

Sparseness and scale-freeness are widely used assumptions. Further evolutionary and functional constraints of large- and medium-scale networks are network motifs, robustness, modularity and evolvability (Marbach et al., 2009[101]). Apart from these generic assumptions, prior knowledge is available and should be exploited from both, databases and literature. Of course, structured information provided in molecular biological databases is preferred over unstructured data in journals and books (see section *‘Integration of prior knowledge’*). 

Currently, both approaches to tackle the problem of dimensionality problem, the extension of measured data and of prior knowledge, may be sufficient for model microorganisms such as the archaeon *Halobacterium* NRC-1 (Bonneau et al., 2006[19]), the prokaryote *Escherichia coli* (Faith et al., 2007[38]; Kaleta et al., 2010[76]) and the eukaryote *Saccharomyces cerevisiae* (Gustafsson and Hörnquist, 2010[55]). For example inferring the genome-wide GRN for *E. coli*, Kaleta et al. (2010[76]). discovered the regulation of the lipoate synthase coding gene (lipA) by the pyruvate-sensing pyruvate dehydrogenase repressor (PdhR). First, they used approximately 1.7 million data points from 380 microarray experiments (76 time series with 5 time points each; N = 4,345 genes). Next, as the number of elements a_i,j_ of the full interaction N x N matrix is 18.9 million and exceeds the number of data, they restricted their study to the interaction of the 316 TFs with their potential target gene. Third, the number of the 1.4 million elements of the TF – target gene interaction matrix was reduced to 878 most significant interactions. Among them, 166 edges (19 %) were already known from the RegulonDB (Gama-Castro et al., 2008[44]). Then, using prior knowledge, in this case study focusing on phylogenetic conserved TF binding sites, the percentage of known TF – target gene interactions could be increased from 19 % to 60 % (65/109). Finally, prior knowledge about the metabolic pathway was exploited to select the *in silico* predicted TF – target gene interaction for experimental validation. In total, 23 new targets of the regulator PdhR were discovered by genome-wide NI (Kaleta et al., 2010[76]; Göhler et al., 2011[48]). This large-scale NI was reliable due to the large number of experimental data and the prior knowledge available in databases, including the database RegulonDB as ‘gold standard’ for assessment of the NI results. 

**Figure 3:**
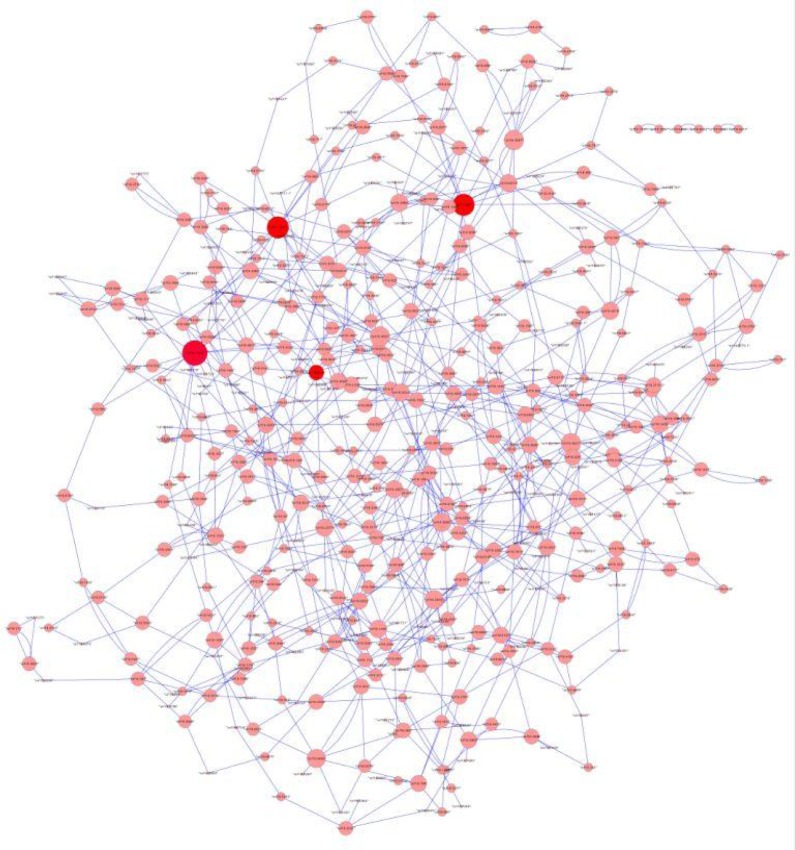
Medium-scale network. 824 interactions inferred using the modified regression method LARS for 503 genes of the ‘gold standard’ of the human pathogenic fungus Candida albicans (Linde et al., 2011, and Altwasser et al., 2012). The red-coloured hubs represent the genes MAL2, SIR2, SNF1 and STE11.

For non-model organisms either experimental data and/or prior knowledge and/or the gold standard are not available in sufficient quantity and/or quality. Thus, genome-wide approaches may lead to GRN of low performance or the performance cannot be assessed. In fact, in most cases the gold standard is simply too small to access performance (as described e.g. for *Staphylococcus aureus* by Marbach et al., 2012[99]). Nevertheless, also in poorly conditioned problems, interesting insights can be gained from medium-scale networks (comprising hundreds of functionally and regulatory characterized genes). Large- and medium-scale networks can also be used to predict potential drug targets and biomarkers for diagnostic purposes and for comparative network analysis (Emmert-Streib et al., 2014[35] and references therein). For instance, large-scale networks (N > 6,000) for the worm *Caenorhabditis elegans* modeling the correlation between differentially expressed genes were used to study changes of global topological parameters, e.g. the mean node degree under different nutritional conditions during aging (Priebe et al., 2013[133]). For the human pathogenic fungus *Candida albicans* hubs of a 503-gene-network were discussed as known and potential targets of antifungal treatment (Altwasser et al., 2012[4]; Figure 3(Fig. 3) (references in Figure 3: Linde et al., 2011[90]; Altwasser et al., 2012[4])).

For genome-wide, large-scale modeling, information theory-based methods (e.g. ARACNE) were found to be applicable, however the LASSO-based regression approaches seem to be superior (Altwasser et al., 2012[4]; Meyer et al., 2014[109]).

### Small-scale networks

In poorly conditioned cases (with respect to the amount of experimental data and prior knowledge), a preferable approach are small-scale networks. The focus is on a subset of genes and proteins and has been demonstrated to be successful for intense interdisciplinary research in biology and medicine. This approach tackles the dimensionality problem by focusing on a subset of genes and proteins, i.e. small-scale modeling instead of the genome-wide approach. The NI of small-scale GRNs is often applied for non-model organisms and tissues. Condensed small-scale GRNs (with up to 50 genes or network nodes) are able to support the experimental design predicting hypotheses of so far unknown mechanisms and interactions in GRNs. Thus, these condensed models could be useful to guide the experimental work (Emmert-Streib et al., 2014[35]). 

The main issue of small-scale GRN inference is the feature selection, i.e. the identification of the ‘most important’ genes or proteins of interest for a certain system or process. For this feature selection there are different approaches. One of them is the clustering of gene expression profiles to select ‘representative’ nodes (D'haeseleer et al., 2000[31]; Wahde and Hertz, 2000[165]; Mjolsness et al., 2000[114]; Guthke et al., 2005[58]). Alternative or complementary approaches focus on certain functional groups of genes and proteins. The functional groups of interest can be selected by identification of differentially expressed genes (DEGs) followed by gene set enrichment analysis (see section *‘Gene enrichment analysis’*). Hypotheses predicted *in silico* using small-scale GRNs were experimentally validated as shown for instance for GRNs describing pathogen-host interaction (Linde et al., 2012[89]; Tierney et al., 2012[157]), adaptation of murine hepatocytes to nutritional change (Vlaic et al., 2012[164]), and mesenchymal stem cell differentiation (Weber et al., 2013[169]). The experimental validation of edges predicted *in silico* by GRN modeling is the best approach for the assessment of NI methods for small-scale GRNs. Here, ODE-based methods such as NetGenerator have been proven to be successfully applicable. Using these tools, hypotheses were predicted in silico and validated experimentally afterwards (e.g. Linde et al., 2012[89]; Tierney et al., 2012[157]; Vlaic et al., 2012[164]; Weber et al., 2013[169]). However, these statements about the performance of NI methods may be biased by the specific application. A more objective and generalizable approach has been performed by the so-called DREAM initiative that will be reviewed in the following. 

In future, multi-scale modeling by merging modular instead of condensed small- and medium-scale models will open the door to a more holistic approach as claimed in systems biology (Sorger, 2005[149]; Ye et al., 2005[175]).

## Assessment of reverse engineering methods by crowdsourcing

In 2006, Stolovitzky, Monroe and Califano initiated the so-called ‘Dialogue for Reverse Engineering Assessment and Methods’ (www.dreamchallenges.org) (Stolovitzky et al., 2007[151]; Prill et al., 2010[135]; Marbach et al., 2012[99]; Bansal et al., 2014[7]). From the DREAM initiative an annual research competition is launched (Table 2(Tab. 2)) and annual DREAM conferences are organized since 2007. Most recently, Califano et al. (2014[22]) reported about the DREAM track of the RECOMB/ISCB Systems and Regulatory Genomics/DREAM Conference 2013.

### Community models achieve high performance

Already as the result of the DREAM2 competition, Stolovitzky et al. (2009[152]) resumed that community models, constructed by aggregating predictions across many models submitted by participants achieve performance on a par with the highest-scoring individual models. Remarkably, this high performance is robust to the inclusion of low-scoring models into the ensemble. That finding is important as no individual NI method has been identified showing best performance for all challenges. The superiority of ensemble learning in GRN inference was confirmed by the following DREAM challenges (Prill et al., 2010[135], 2011[136]; Marbach et al., 2010[102]; Margolin et al., 2013[103]). Also, summarizing the results of evaluating the GRN inference methods within the DREAM5 competition (Challenge 4 in 2010; Table 2(Tab. 2)), Marbach et al. (2012[99]) stated that ‘the collective knowledge of a community is greater than the knowledge of any individual’. Consequently, methods of combining the information contained within an ensemble of inferred networks were developed (Marbach et al., 2009[100]).

The goal of the ‘DREAM5 – Challenge 4’ (Table 2(Tab. 2)) was to infer an in silico benchmark network model (1,643 genes) as well as genome-scale GRN from gene expression microarray datasets for the well-studied microorganisms *E. coli* (4,297 genes, 805 arrays), S. aureus (2,677 genes, 160 arrays) and *S. cerevisiae* (5,667 genes, 536 arrays). The evaluation of the *S. aureus* network was excluded from comparison because there are enough experimentally supported interactions (‘Gold standard’) for network validation available. A total of 35 methods for GRN inference were applied and compared, including regression (8 methods), mutual information (5), correlation (3), Bayesian (6) and other (12) methods as well as combinations of them. The main conclusions from this evaluation of GRN inference methods for genome-scale GRN are the following (Marbach et al., 2012[99]):

(i) After excluding *S. aureus*, the quality of the GRN for *S. cerevisiae* was lowest independent of the applied inference method due to the highest number of genes and relative low number of data. 

(ii) Best performance and highest robustness (against poorly performing inference methods) were obtained by community NI approach. In particular, this approach should be preferred for non-model organisms such as S. aureus with scarce prior knowledge. 

(iii) In general, BN inference methods were outperformed by regression and mutual information approaches (for both *E. coli* and *in silico* GRN). It is known that BN need more data than other NI methods. Integration of prior knowledge, which is an important advantage of BN, was not requested in the ‘DREAM5 – Challenge 4’. 

(iv) Certain regression methods show a performance similar to the best-performing community NI. The well-established mutual information NI methods CLR and ARACNE are outperformed by certain LASSO/LARS-based regression methods. The method TIGRESS (Haury et al., 2012[64]) combined LARS with a novel feature selection method (‘stability selection’). However, LASSO combined with bootstrapping, which was found to be the best performing individual method for the *in silico* NI, achieved only a low score for the *E. coli* GRN. 

(v) A specific inference method (Küffner et al., 2012[80]) outperformes the community NI – but only for the *E. coli* GRN inference: Here, co-dependencies between TFs and target genes are detected by two-way ANOVA method. In addition, TF perturbation data are up-weighted. For the *E. coli* NI also very good results were obtained using the LASSO toolbox GENLAB with default parameters (http://genlab.tudelft.nl/genlab.html; van Someren et al., 2006[161]).

(vi) The random forest-based method GENIE3 was the best performer in the ‘DREAM4 – Challenge 2’ for *in silico* NI inference (Huynh-Thu et al., 2010[70]) and reached also a high score in the DREAM5 – 4 challenge for both *in silico* and *E. coli* NI. The random forest-based method GENIE3 was ranked with highest overall score in the evaluation of the ‘DREAM 5 - Challenge 4’ (Marbach et al., 2012[99]). Here, decision trees are used to produce prioritized lists of TFs regulating each target gene.

### Integration of heterogeneous data 

Both, the ‘DREAM7 – Challenge 1’ and the ‘DREAM6 – Challenge 2’, aim to evaluate methods for model structure discrimination and for the estimation of parameters in non-linear biochemical models that characterize the dynamics of molecular processes (Meyer et al., 2014[109]). In the ‘DREAM7 – Challenge 1’, an 9-gene network composed *in silico* was used as gold standard for parameter estimation. Additionally, an incomplete 11-gene network model was used as gold standard to assess methods for identification of three missing links. A virtual budget was provided in this challenge to ‘buy’ experimental data generated (*in silico*) by model simulations. The expense of different experimental techniques, such as transcriptome profiling measured by microarrays as well as abundance of all proteins measured by mass spectrometry, both with low and high temporal resolution (500 and 100 credits, respectively) and protein abundance for 2 proteins of choice with highest temporal resolution (for 400 credits) was mimicked. Furthermore, perturbation experiments, such as knock-out, knock-down and decrease of ribosome binding site experiments are offered for choice (for 800, 350, and 450 credits, respectively). The analysis of the results from 19 competing teams suggests that the combination of state-of-the-art parameter estimation and a varied set of experimental methods using a few datasets, mostly proteome (fluorescence imaging) data, can accurately determine parameters of biochemical models of gene regulation. 

The identification of the missing links in the incomplete 11-gene network was more challenging. For identifying the missing links of the 11-gene network, the best-performing team first used credits on wild type fluorescence data, to cheaply obtain a setting with qualitative disagreement between data and model, and then used mass spectroscopy experiments with perturbations to test for potential missing links. For discrimination between the alternative model structures, Meyer et al. (2014[109]) applied classical maximum likelihood methods. However, only the consensus obtained by majority voting to select the most submitted links had a top performing score. Only one of the three consensus links was correctly inferred, while the direction and nature of the regulatory link of the two others were incorrect. This demonstrates the difficulty to correctly identify the topology also of small-scale GRNs based solely on limited experimental data and perturbations. Thus, integration of prior knowledge is indispensable also for small-scale GRN inference. Again, it was found that aggregating independent parameter predictions and network topologies across submissions created a solution that can be better than the one from the best-performing submission (Meyer et al., 2014[109]).

The DREAM challenges were not only focused on the assessment of GRN inference methods. As the consequence of the conclusion that genome-wide GRN modeling is reliable only for well-conditioned problems and that the identification of the GRN topology may be wrong also for small-scale GRN (if no prior knowledge was included), more simplified but useful computational problems were addressed by the DREAM competition. Thus, there were also challenges to evaluate methods for gene expression and biomarker prediction as well as to assess the performance of classifiers for diagnosis of diseases. Ruan (2010[140]) showed for the prediction of gene expression values that the simple k-nearest-neighbor method led to almost the same performance as a much more sophisticated method. 

In the following, three examples from DREAM7 will be discussed that provide recommendations for inference of predictive models in a clinical perspective.

In the ‘DREAM7 – Challenge 2’ (Table 2(Tab. 2)), the results of breast cancer survival prediction by more than 1,400 computational models from 354 research groups were evaluated. The models were trained on the data set of 1,000 samples including clinical information (for example, age, tumor size and histological grade), mRNA expression data and DNA copy number data. The models were validated on data sets of 981 samples. The predictive value of each model was scored by calculating the concordance index (CI) of predicted death risk. In a final phase, the data of all 1,981 samples were used for model refinement and the retrained models were validated on a further data set from 184 women diagnosed with breast cancer. The best-performing model combined clinical features and molecular features selected by prior knowledge. A machine learning method (boosted regression) was applied to a combination of clinical features, expression levels of genes selected by data-driven criteria and by their involvement in breast cancer (Ravasi et al., 2010[139]) and, finally, an aggregated “genomic instability” index calculated from the copy number data (Bilal et al., 2013[16]). Margolin et al. (2013[103]) found that the top-scoring models used a methodology that minimized overfitting to the training set by defining a “Metagene” feature space based on robust gene expression patterns observed in multiple external cancer data sets. Long-time survivors are better predicted than short-time survivors.

The goal of the ‘DREAM7 – Challenge 3’ was to predict the future progression of disease in Amyotrophic Lateral Sclerosis (ALS), a neurodegenerative disease. The results of 37 algorithms were submitted for evaluation. Interestingly, the two best algorithms outperformed predictions by ALS clinicians. Küffner et al. (2015)[81] estimated that using both winning algorithms in future trial designs could reduce the required number of patients by at least 20 %. In addition, several potential non-standard predictors of ALS progression were identified including uric acid, creatinine and blood pressure. Thus, this DREAM challenge contributed to a better understanding of ALS pathobiology.

The ‘DREAM7 – Challenge 4’ dealt with prediction of the drug response from multi-omics data (CNV, RPPA, Methylation, Exome sequencing, gene expression microarray, RNA-seq) measured in human breast cancer cell lines, a total of 44 drug sensitivity prediction algorithms were analyzed (Costello et al., 2014[28]). The best results were obtained using nonlinear relationships and incorporating the biological pathway information. In addition, they found that gene expression microarrays provided the best predictive power, however, the performance increased including further data sets. The top-3 approaches used Bayesian multitask Multiple Kernel Learning, weighted nonlinear regression trees and weighted features from Pearson’s correlation.

## Challenges

### Integration of prior knowledge

The prediction of GRNs is a great combinatorial challenge usually based on a limited amount of data. Some inference tools integrate prior knowledge to support the inference process. Supportive interaction knowledge is usually of positive nature, meaning that the existence of an interaction was predicted or experimentally observed. Negative prior knowledge about non existing interactions is equally important for network inference, but hardly available. One reason is that the experimental proof that two genes never interact is very hard to do. Some journals publish negative results (Journal of Negative Results in Biomedicine; New Negatives in Plant Science (Elsevier); Journal of Negative Results – Ecology & Evolutionary Biology) from which negative prior knowledge can be extracted. Nevertheless, to our knowledge no databases exist that allow easy access to negative prior knowledge. 

There is an increasing number of molecular biological databases. Currently, the Nuclear Acid Research Online Molecular Biology Database Collection has been expanded to 1,552 databases (Fernández-Suárez et al., 2014[39]). Olsen et al. (2014[125]) assessed the relevance of different prior knowledge sources for inferring GRNs in cancer research. The most direct interactions that can be obtained as prior knowledge for GRNs are TFs interacting with promoters. Promoters can be analyzed for known TF binding profiles (free JASPAR database (Mathelier et al., 2013[106]), commercial TRANSFAC database (Matys et al., 2006[107])) or motifs can be elicited (e.g. MEME (Bailey et al., 2009[6])). Experimentally, TF-DNA interactions are determined by ChIP-Seq resulting in p-values of interactions. These p-values are inversely correlated to the probability of an edge being present in a GRN (Bernhard and Hartemink, 2005[15]).

Various databases have been established to provide knowledge about genes and interactions. Some include many organisms (e.g. STRING (Szklarczyk et al., 2015[153])), others are species specific such as the Candida Genome Database (Binkley et al., 2014[17]). Over the last years, pathogen-host interaction databases emerged and are listed in Mukherjee et al. (2013[119]). One example is PHISTO (Tekir et al., 2013[155]), which includes >16,000 virus-host interactions, > 8,000 bacteria-host interactions but only few fungus-host interactions (<10). Especially for not well studied organisms available knowledge can be limited and insufficient. One option would be to extract knowledge of homologous genes in closely related organisms.

Human database curation teams cannot keep up with volume and pace of literature production (Baumgartner et al., 2007[14]). Thus, text mining needs to close this gap by extracting structured knowledge from unstructured information such as scientific literature (Hahn et al., 2007[60]). Text-mining tools as for instance the commercial Pathway Studio for mammals (Nikitin et al., 2003[124]) or the free Gene Interaction Miner (Ikin et al., 2010[72]) facilitate automatic knowledge extraction from literature databases. For well studied organisms this can result in a lot of interactions, for which manual curation might not be feasible anymore. 

Furthermore, more knowledge is available for well studied genes which has to be taken into account when predicted GRNs are interpreted. Also, web applications exist that retrieve knowledge from various sources (Haibe-Kains et al., 2012[61]; Horn et al., 2014[68]). Given a set of genes, the web platform GeneMANIA (Mostafavi et al., 2008[118]; Montojo et al., 2014[115]) queries biological databases, published articles and co-expression from published data sets. It returns a network of interactions distinguishable by source and is available for *H. sapiens* and eight model organisms (e.g. *Mus musculus, S. cerevisiae*). 

### Pathogen-host GRNs

Next Generation Sequencing techniques paved the way for advanced genomic and transcriptomic studies of interacting species, in particular for metagenomic and infection research (Pallen et al., 2010[127]). Dual RNA-Seq is an approach, where transcriptomes of two or more species are sequenced together. Westermann et al. (2012[171]) reviewed various aspects that have to be considered when dual RNA-Seq samples are prepared and sequenced. 

One application of dual RNA-Seq is to study pathogens interacting with their host, e.g. filarial worm – mosquito interactions (Choi et al., 2014[25]) and Azospirillum brasilense colonizing wheat roots (Camilios-Neto et al., 2014[23]). Furthermore, Tierney et al. (2012[157]) published a dual RNA-Seq time series data set of mouse dendritic cells infected with *C. albicans*. Six pathogen genes and five host genes were selected and the small-scale NI tool NetGenerator was applied. Two of the predicted interactions were experimentally validated demonstrating the applicability of GRN inference to model pathogen-host interactions. Recently, NetGenerator was extended and its application to infer pathogen-host GRNs was outlined (Schulze et al., 2015[145]). The focus was on accounting for pathogen-host interaction data characteristics, such as changing environmental conditions, temporally different onsets of transcriptional responses and possible missing data points (e.g. only one organism survives). Furthermore, they give an overview of basic requirements and main steps of acquisition and analysis of dual RNA-Seq data.

### Non-linear models

Based on fundamental knowledge from thermodynamics of irreversible processes and self-organization (Prigogine and Nicolis, 1971[134]) it is known that living systems have to be modeled using non-linear functions. However, adequate non-linear modeling requires more experimental data or/and prior knowledge, which is in most cases already insufficient for linear modeling (problem of dimensionality). The DREAM2 challenge provided an *in silico* network with 50 genes and a ‘comfortable’ set of 50 x 26 x 23 data without noise and the result showed that also the best-performing LASSO-based NI with a set of non-linear basis functions does not correctly infer the non-linear relations (Gustafsson et al., 2009[57]). To include non-linearity in the GRN but apply the benefits of linear modeling, piecewise linear models were proposed (Westra et al., 2011[172]), but they are not extensively studied for GRN inference so far. Thus, linear models are often applied despite the knowledge that the assumption of linearity is not ‘true’. Linear models may be wrong, but useful for prediction of hypotheses (e.g. regulator – target gene relations) or the behavior of the system close to the steady state (e.g. outcome of a disease). However, in general, such modeling is insufficient to predict a molecular mechanism in detail and for simulation of the dynamic behavior with multiple attractors (e.g. multiple steady states; Milnor, 1985[113]). Small- and medium-scale models are helpful to support and design experiments. To overcome the reductionistic view and go ahead to a more holistic one, multi-scale modeling merging various validated small-scale models will be the future in systems biology of gene expression network modeling.

### Model validation

Most important, GRN results have to be validated experimentally. Typically, this is an iterative process in systems biology forming a cycle of wet-lab and dry-lab research (Figure 1(Fig. 1)). Based on experimental data and prior knowledge at hand, an initial network model can be inferred. Then, hypotheses have to be generated based on the draft model and should be experimentally tested or checked by literature search. In general, this experimental validation or deep literature search will give rise to refined network modeling. For this second round of network modeling, the results of experimental validation or literature search can be included as improved prior knowledge. Of course, some prior knowledge relations can be contradictory to each other or to the experimental data. Contradictory prior knowledge can be handled by ranking it with a score according to the experimental method and setup used for drawing the respective conclusion. The score can also reflect how close the experimental setup of the referenced system is to the currently studied system (with respect to experimental conditions, tissues, organisms). Such scoring systems are in its infancy (e.g. Linde et al., 2010[90]).

## Conclusion

The mathematical and computational modeling of networks is of great importance in biomedical research to understand molecular mechanisms, e.g. pathogen-host interaction. To tackle the complexity inherent in large networks of interacting biomolecules, many different approaches and methods have been established. They include methods based on Boolean networks, Bayesian Networks, information theory, differential or difference equations, graphical Gaussian models and/or supervised machine learning methods. In general due to the high dimensionality (thousands of genes and proteins) versus the limited number of samples (not more than hundreds), the GRN inference is underdetermined implying that there could be many equivalent (indistinguishable) solutions. To cope with this fundamental problem, there are various approaches for GRN inference. Some of them are widely used and powerful, such as the information theory-based methods (like the ARACNE or Context Likelihood of Relatedness – CLR method) and the regression-based LASSO method for large-scale network models, whereas ODE-based methods, such as Inferelator and NetGenerator, for dynamical medium- and small-scale models, respectively. During the last decade the scientific community improved the understanding when and how to apply them. As a trend of the last five years, different methods of NI and different data types, including prior knowledge, were combined and integrated to improve the performance.

First of all, no individual GRN modeling approach performs best for all problems. The DREAM competition showed that ensemble learning, i.e. ‘community models’ constructed by aggregating predictions across many models, allows NI with high performance and robustness against the inclusion of low-performance models. Integration of predictions from multiple inference methods is robust and has high performance across diverse datasets (Marbach et al., 2009a[100]; 2012[99]). For instance, the algorithm TRaCE performs an ensemble inference of GRNs (Ud-Dean and Gunawan, 2014[160]).

Second, NI should not be based only on the limited number of gene expression data. GRN inference should integrate prior knowledge (see section *‘Integration of prior knowledge’*) or further, heterogeneous experimental data sets (Greenfield et al., 2010[51]). In particular, GRN inference using gene expression data should be supported by information about already known TF – target gene interaction, TF binding sites or their motifs, composites of TFs and signaling pathways from receptors to TFs. Marbach et al. (2009[101]) won the synthetic five-gene network challenge of the reverse engineering competition inference DREAM2 by integration of prior knowledge mimicking the evolutionary process.

Third, to infer networks with directed edges, data should be exploited that represent cause–effect relations. Typically, these are time series data of response to known perturbations (interventions), steady state data from knock-out (KO) experiments or assigned with Singular Nucleotide Polymorphism (SNP) or other genotype data. The type of these data is essential for the choice of inference method. For small-scale network modeling using time series data and prior knowledge, ODE-based inference tools, such as NetGenerator (Weber et al., 2013[169]), should be preferentially applied. For medium-scale models based on time series and mixed data including steady state data, the ExTILAR algorithm (Vlaic et al., 2012[164]) was developed. For large-scale network modeling using time series data, TimeDelay-ARACNE is applicable (Zoppoli et al., 2010[182]). However, for genome-wide and other large-scale networks the regression-based method LASSO (GENLAB, van Someren et al., 2006[161]) seems to be best situated if it is well configured and the experimental data and prior knowledge are of sufficient quantity and quality (Marbach et al., 2012[99]). Boolean and static network modeling should be preferred if the data are mainly steady state gene expression data from KO experiments or for modeling of signaling pathways, respectively (Eduati et al., 2010[33]; Klamt et al., 2010[78]; Flassig et al., 2013[41]; Samaga and Klamt, 2013[142]; Pinna et al., 2013[129]; Ryll et al., 2014[141]; Nakajima and Akutsu, 2014[121]).

Today, large-scale models for model organisms (e.g. *E. coli* and *S. cerevisiae*) reflect some general properties such as the robustness and stability of the system or they give information about hubs, e.g. most important transcriptional regulators and target genes. However, they do not comprehensively represent all interactions and their dynamics. Currently, for non-model organisms, small-scale networks with a certain focus, is a useful approach. However, we expect that in future also for non-model organisms genome-wide GRN models with improved predictive power will be established based on extended experimental data compendia and molecular interactome databases.

## Acknowledgements

This work was supported by the Deutsche Forschungsgemeinschaft (DFG) CRC/Transregio 124 ‘Pathogenic fungi and their human host: Networks of interaction’, subproject INF (JL, RG) and B3 (SS, RG). SGH was supported by the German Federal Ministry of Education and Research (BMBF) within the Virtual Liver initiative.

## Figures and Tables

**Table 1:**
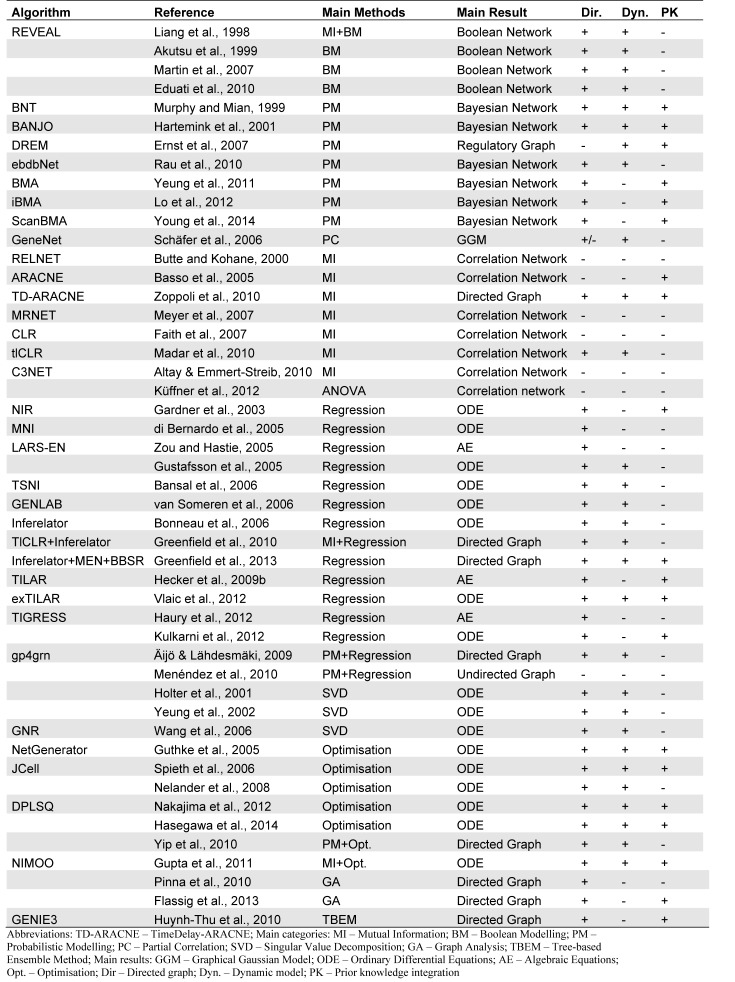
GRN inference methods

**Table 2:**
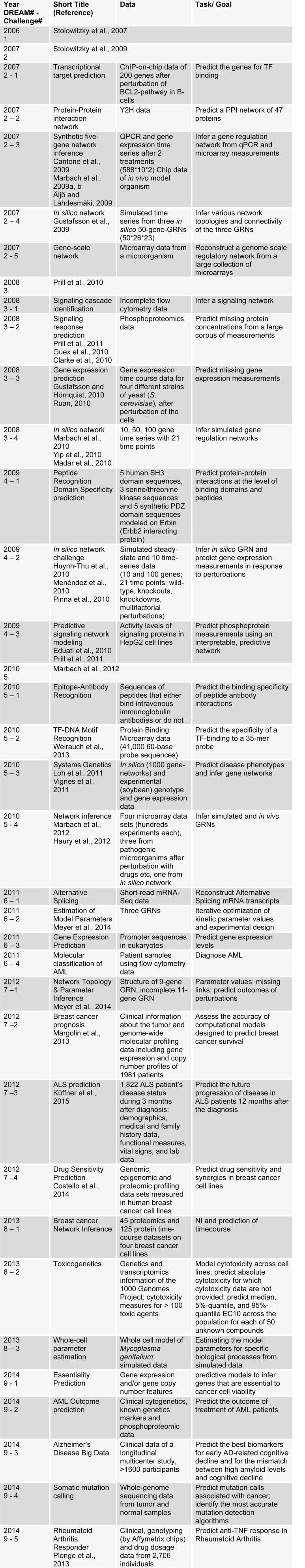
DREAM Challenges. DREAM#: Running number of challenge; Short Title and reference if any; Data given for the challenge
